# Reducing Iron Oxide Content in Phosphoric Acid Using Polyacrylates and Phosphonic Acids

**DOI:** 10.1021/acsomega.5c05371

**Published:** 2025-11-25

**Authors:** Gustavo Paiva Ribeiro, Sandra Cristina Dantas, Eloízio Júlio Ribeiro, Carla Eponina Hori

**Affiliations:** † Faculdade de Engenharia Química, 28119 Universidade Federal de Uberlândia, Avenida João Naves de Ávila 2121, Bloco 1 K, Campus Santa Mônica, Uberlândia, Minas Gerais CEP 38408-144, Brazil; ‡ Departamento de Engenharia Química, 74348 Universidade Federal do Triângulo Mineiro, Avenida Randolfe Borges Júnior 1400, Univerdecidade, Uberaba, Minas Gerais CEP 38064-200, Brazil

## Abstract

Phosphoric acid is the second most produced and consumed inorganic acid in the world and the most important intermediary in the production of phosphate fertilizers. The quality and agronomic utilization of phosphate fertilizers are highly dependent on the levels of contaminants present on the phosphoric acid, especially iron oxides. The presence of iron oxides in phosphate fertilizers results in the formation of iron phosphates possessing lower agronomic efficiency and a reduced commercial value. The wet-process phosphoric acid uses phosphate rocks or concentrates as a source of P_2_O_5_. Most phosphate rocks in Brazil originate from igneous mines, with high concentrations of iron oxides, as opposed to most mines in operation in the world, with sedimentary origins and lower contaminant contents. Iron content is a limiting factor in the economic and technical feasibility of phosphate mines, and the complete removal of iron oxides during the industrial concentration of phosphate rock is unfeasible due to phosphate losses. To improve the quality of the phosphoric acid produced from such ores and to enable the usage of phosphate rock concentrates with higher levels of contaminants, this study aimed to evaluate the effectiveness of two compounds for removing the iron oxides from the acid: (i) a mixture of polyacrylates associated with phosphonic acids and (ii) a solution with 60% of diethylenetriamine penta (phosphonic methylene) (60% DTPMP). The mixture of polyacrylates associated with phosphonic acids showed limited effectiveness in removing contaminants from the phosphoric acid. The DTPMP solution, however, proved effective as an iron chelating agent in phosphoric acid, whereas the Fe_2_O_3_ content on a dry basis was reduced by 40% and the P_2_O_5_/Fe_2_O_3_ ratio was increased by 59%. The optimal reactional conditions were *T* = 55 °C, 14 h of precipitation time, and 5.57% of chelating reagent in relation to the initial phosphoric acid mass (w/w). A total of 0.58% of Fe_2_O_3_ was removed from the acid, a yield comparable to the 0.55% observed in other benchmark studies.

## Graphical abstract

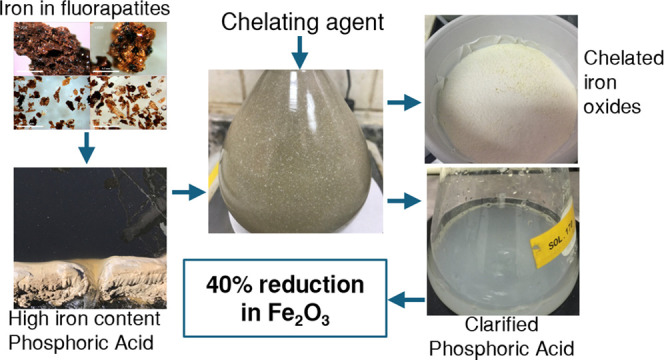

## 1 Introduction

One of the Sustainable Development Goals set by the United Nations is to ensure food security while shifting toward sustainable agricultural practices.^1^ Brazil is a country with a great vocation for agribusiness, and in recent years, more than 20% of its Gross Domestic Product has come from this sector.^2^ However, Brazilian soils have generally low concentrations of nutrients essential to plant growth, among them phosphates (P_2_O_5_), which must be supplemented by soil fertilization.^3,4^ There are several phosphate-based fertilizers available on the market, and basically all high-grade options use phosphoric acid as an intermediate product or ingredient.^5–8^

Approximately 90% of the phosphoric acid produced in the world uses a production route denominated “Wet Process Phosphoric Acid” (WPA), which consists of the reaction of phosphate rocks or phosphate concentrates with sulfuric acid and water, yielding phosphoric acid and calcium sulfate as main products.^9^ However, phosphoric acids produced by the wet route retain most of the impurities present in the phosphate concentrates or rocks used in their production.^10^ Therefore, the phosphate rock or concentrate used affects the composition, characteristics, and potential use of the produced phosphoric acid. For all economical and practical purposes, the phosphate rock is the main source of P_2_O_5_ for most phosphates’ fertilizers and phosphoric acid.^10,11^

Most phosphate rocks used in Brazil possess low P_2_O_5_ content and originate from igneous mines due to the country’s geological characteristics.^12^ The formation of these mines resulted in extremely complex mineralogies, where apatite [Ca_5_(PO_4_)_3_(OH, F, Cl)], the primary source of phosphorus on igneous phosphate rocks, coexists with impurities such as silica, iron and aluminum oxides, barium oxides, sulfates, titanium, magnesium, and others.^13,14^ One of the primary contaminants in igneous mines is iron oxide, typically expressed as Fe_2_O_3_, but chemically present in the forms of hematite, magnetite, ilmenite, and goethite, among others. The presence of iron oxides in the solubilization of apatites when producing fertilizers or phosphoric acid leads to the formation of iron phosphates, which are insoluble in water, partially insoluble in neutral ammonium citrate, with lower agronomic efficiency for most crops.^15^

With increasing levels of iron oxides, igneous deposits in Brazil generate phosphoric acids with high levels of iron phosphates. The usage of such acids may result in products outside their chemical and physical specifications due to insolubilization or dilution of the P_2_O_5_ content required and may even render mining operations unfeasible due to quality specifications, legal requirements, or cost in complementing extra P_2_O_5_ content. There is also a noticeable impact when producing higher-grade fertilizers, such as diammonium phosphate and monoammonium phosphate (MAP). Since iron phosphates do not react with ammonia, the lack of free phosphoric acid generates products where the nitrogen specifications are not met.^9^

Consequently, methods must be developed to mitigate the effects of iron oxides present in phosphate concentrates or iron phosphates present in phosphoric acid, aiming at the sustainability of the national industry, especially during a period when there is a global increase in fertilizer demand.^16,17^ The usual method for reducing the effects of the levels of iron oxides present is their removal in the processes of mineral concentration; however, such routes have technical and economic limitations.^9,18^ One possibility is developing methods for eliminating iron oxides from the phosphoric acid itself, as almost all contaminants are soluble in the liquid phase. Such routes have inherent advantages when compared to their removal from phosphate concentrates since the contaminants present in the acid have a relatively uniform composition. Therefore, chelation and precipitation mechanisms for iron oxides can be applied, since the process routes for phosphoric acid necessarily include steps to clarify the acid, as well as a filtration operation for gypsum removal.^19,20^

The main advantages of precipitation techniques applied for phosphoric acid purification are their low cost, which is dependent only on the price of the precipitation reactant, and the fact that WPA processes already possess the necessary separation equipment to handle precipitates and solids. The main disadvantage of precipitation methods is P_2_O_5_ losses due to coprecipitation along with the precipitated metal components. So, several works focusing on finding reagents that can be used for purification of phosphoric acid with low or minimum P_2_O_5_ losses were carried out in the past years, some even trying conditions that could allow the regeneration of the reactant added or the recovery of precipitated P_2_O_5_.^20–22^ However, most of these studies are limited to phosphoric acid derived from sedimentary phosphate rocks, which have lower iron contents and different impurity profiles. Although significant progress has been made with those sources, few investigations have addressed the specific challenges associated with phosphoric acid obtained from igneous ores, particularly those processed in Brazil, where iron contamination is more pronounced and complex.

The present work has as its objective to reduce the iron content of phosphoric acid produced from igneous rocks by the usage of two reagents: (i) a solution of polyacrylates associated with phosphonic acids and (ii) an acid solution of 60% diethylenetriamine penta (phosphonic methylene) (60% DTPMP), in a concept where the diluted phosphoric acid is reacted with the reagents on conditions defined on a statistical planning, and the precipitate formed is directed toward the gypsum filters, so it can be washed and stacked along the gypsum. Reaction conditions that could increase the iron oxide removal while maintaining or minimizing P_2_O_5_ content losses were studied. This work was conducted in an industrial site that is a key producer of phosphoric acid and phosphate fertilizers located in southeastern Brazil, with the focus of reaching an industrial solution to a chronic problem. The experiments were conducted on a bench scale, in premises to allow the eventual scale-up of the route to pilot and industrial scales.

## 2 Experimental Section

### 2.1 Phosphoric Acid Sample

A 1000 kg portion of diluted industrial phosphoric acid was collected from a single batch produced on an industrial site using a blend of two phosphate concentrates of igneous origin. The sample was collected after the clarification step, employed to reduce the content of precipitated CaSO_4_. The sample was transported in a thermally insulated container to maintain a constant temperature during the transit from the industrial unit to the laboratory. Subsequently, the sample was homogenized, characterized, fractioned, and stored in a laboratory oven with a controlled temperature set at 60 °C throughout the entire duration of the experiments.

### 2.2 Iron Removal Experiments

#### 2.2.1 Iron Removal Reactions

The experiments to evaluate iron removal in diluted phosphoric acid were conducted by considering the following variables: X1, reaction temperature; X2, precipitation time; and X3, percentage of reagent mass added relative to the phosphoric acid mass. Further details regarding the selection of values are provided in the experimental design section. For each experiment, 500 g of diluted phosphoric acid, previously stored at 60 °C, was used. A thermostatic bath was set to the reaction temperature (X1). Once the defined temperature was achieved, the test reagent (polyacrylates associated with phosphonic acids or 60% DTPMP) was added (X3) to the phosphoric acid under agitation of 350 rpm.

Following 60 min of agitation, the mass of the reacted phosphoric acid was measured to calculate the water evaporation rate in the acid. Each batch produced was stored in a hermetically sealed container and left undisturbed in a temperature-controlled environment for a specific time (X2). After the resting period, each container was homogenized, opened, and completely filtered through a white strip paper filter under a vacuum.

After the complete filtration of the samples, the cake was washed with 100 mL of distilled water and filtered, and the filtrate was analyzed to evaluate the phosphoric acid and other soluble components present in the filtrate. The washing step is meant to simulate the washing the cake will incur during the industrial process, as the formed cake will be washed along with the gypsum on an industrial route to minimize soluble P_2_O_5_ losses. The water volume used to wash the cake was defined as proportional to the mass used in the industrial route. Following the water wash, the cake was rinsed with 200 mL of acetone to eliminate any traces of phosphoric acid and to facilitate drying at low temperatures, thus allowing the solids to be characterized.

After the cakes were dried, the P_2_O_5_ and Fe_2_O_3_ levels in the clarified phosphoric acids were analyzed. The P_2_O_5_, Fe_2_O_3_, MgO, CaO, and Al_2_O_3_ contents on the generated precipitates were analyzed, along with the total P_2_O_5_ and Fe_2_O_3_ contents in the washing water. Analyses of the washing water filtrate were conducted not only to quantify the P_2_O_5_ and Fe_2_O_3_ balance in the system but also to indirectly assess the filtration rate of the generated cakes. Higher P_2_O_5_ contents on the wash water would indicate a smaller specific surface area of the solids generated, potentially resulting in a reduction of the filtration rate, with possible consequences for the productivity of industrial units. Figure 1 illustrates the procedure described.

**Figure 1:**
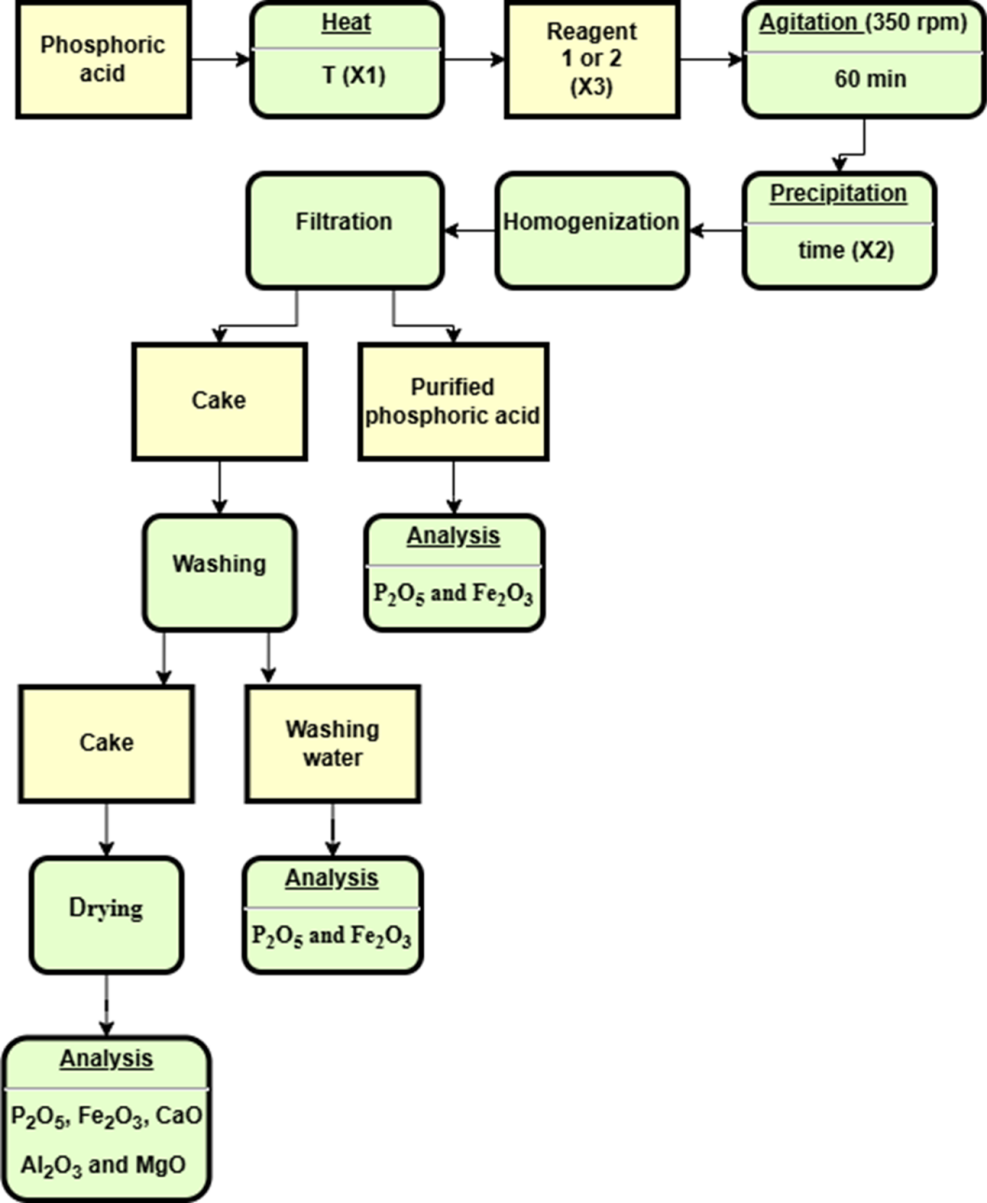
Flow diagram of phosphoric acid with iron removal process.

#### 2.2.2 Analytical Methodologies for Characterization

Throughout all stages of the conducted experiments, laboratory methodologies were employed to characterize the phosphoric acid batches as well as the resulting clarified acid and precipitated solids. The analytical methods utilized were developed based on the “Manual of Official Methods for Mineral, Organic, Organomineral, and Corrective Fertilizers”,^12,22,23^ adapted for the industrial routine. All analyses were carried out in a chemical laboratory within a production complex specialized in the production of phosphoric acid and phosphate fertilizers and are listed below.

The methodology for analyzing P_2_O_5_ levels involves preparing a dye solution and employing colorimetric analysis. Similarly, the determination of the P_2_O_5_ content in precipitates and gypsum follows a comparable procedure. To determine the iron content, the sample was decomposed with hydrochloric acid, where the trivalent iron is reduced with stannous chloride and the excess is eliminated with mercuric chloride. Reduced iron is titrated by potassium dichromate using barium diphenylamine sulfonate as an indicator. The analysis of calcium oxide content is based on the precipitation of calcium in the form of calcium oxalate, which is dissolved in sulfuric acid and subsequently titrated with potassium permanganate. The analysis of magnesium oxide contents is based on the principle of atomic absorption, where the MgO contents of the samples were analyzed through the methodology described in NBR 8538.^24^

#### 2.2.3 Design of Experiments

Central Composite Designs (CCD) developed by Box and Wilson^25^ were used in this work. The CCDs were designed to present an orthogonality α of 1.2872 with two central replicas, aiming at creating feasible intervals within an industrial process (Table 1). The following independent variables were studied in this work: temperature (°C) (*T*), precipitation time (hours) (*t*), and percentage of mass of reagent in relation to mass of acid (%). The variable levels were defined according to operational values possible for each variable on the industrial site at which the work took place, as a basic premise of this work was to be able to industrially reproduce the trials and optimum conditions without the need to create a new process route requiring additional equipment or capital expenditures.

**Table 1:** Experimental Design Variable Levels for the Iron Removal Reactions

		level				
variable	nomenclature	–α	–1	0	+1	+α
temperature (°C)	X1	22.82	30	55	80	87.18
precipitation time (hours)	X2	1.13	4	14	24	26.87
percentage of mass (%)	X3	0.43	1	3	5	5.57

The central point of variable X1 is the usual temperature of the phosphoric acid during clarification, while the +1 level is the usual temperature of the phosphoric acid when it is exiting the reactor on the dehydrate route. X2 central point was defined as the average residence time used on the phosphoric acid clarification, while X3 was defined by the cost of the reactants. Although it is known that a higher dosage of reagent will likely reduce the iron content present in phosphoric acid until an excess is reached, such dosage increases would not be economically viable, and thus, the variable X3 was limited so that the solution could be cost-effective.

Two CCDs were carried out: the first using a solution of polyacrylates associated with phosphonic acids (CCD 1) and the second using a solution of 60% DTPMP (CCD 2). For both CCD, the P_2_O_5_/Fe_2_O_3_ (w/w) ratio in the phosphoric acids produced was considered as the main response to the experiments. The usage of ratios between acid components is a common practice in the WPA process route as it eliminates the need to consider water evaporation during the various processing steps. Also, it eliminates the need to carry out a water mass balance for each experiment, since the reaction temperature was a variable, leading to different water evaporation rates. By making this correlation, the levels of P_2_O_5_ and contaminants increase or decrease on equivalent proportions independent of the sample water content. Therefore, increases in the P_2_O_5_/Fe_2_O_3_ ratio on phosphoric acid indicate that more iron oxides were removed from the acid by the reaction. The industrial specification of the P_2_O_5_/Fe_2_O_3_ ratio varies according to the final fertilizer specifications, and a usual benchmark for this ratio is a minimum of 20 when considering the production of MAP with 11% of nitrogen and 52% of P_2_O_5_ on its composition, which is the main product produced on the manufacturing site where the work was conducted. The other responses were all based on the content of P_2_O_5_, Fe_2_O_3_, CaO, Al_2_O_3_, and MgO, as well as the total mass generated as a solid precipitate.

The CCD coupled with response surface methodology allows an evaluation of the influence of the parameters related to each independent variable on the responses of the process.^26^ Regression analysis was used to obtain equations to predict each yield as a function of the independent variables. The significances of the parameters were tested using an analysis of variance. The adequacy and quality of fitting were evaluated by the coefficient of determination (*r* ^2^) and residual analyses. All statistical analyses were conducted using Statistica 10.0.

## 3 Results and Discussion

### 3.1 CCD 1—Mixture of Polyacrylates Associated with Phosphonic Acids

The first central composite design, called CCD 1, used as a reagent a proprietary chelating mixture, produced based on various phosphonic acids and polymers based on polyacrylates. Table 2 shows the P_2_O_5_ and Fe_2_O_3_ contents of phosphoric acid for all points of CCD 1, as well as the contents of the reference test. Due to the variation observed by the moisture loss, the results are better represented by the ratios between the P_2_O_5_/Fe_2_O_3_. It is noted that all tests resulted in an increase in relation to the reference test, indicating that the use of polyacrylates associated with phosphonic acids resulted in a decrease in the iron content in phosphoric acid. The lowest iron oxide content was obtained in run 2, representing a 10.3% decrease, and the highest P_2_O_5_/Fe_2_O_3_ ratio was 18.42, achieved in run 14, which used the largest amount of reagent 1. Even though an increase in the P_2_O_5_/Fe_2_O_3_ ratio was achieved, the results failed to reach a specification ratio of 20, a benchmark required for adequate MAP production.

**Table 2:** P_2_O_5_ and Fe_2_O_3_ Contents in Phosphoric Acid Obtained Using Polyacrylates Associated with Phosphonic Acids as Additive (CCD 1)

CCD 1	variables			response		
	X1	X2	X3	P_2_O_5_ (%)	Fe_2_O_3_ (%)	P_2_O_5_/Fe_2_O_3_
reference	-	-	-	23.86	1.46	16.34
1	–1	–1	–1	24.22	1.39	17.42
2	–1	–1	1	23.70	1.31	18.09
3	–1	1	–1	24.25	1.44	16.84
4	–1	1	1	23.66	1.32	17.92
5	1	–1	–1	30.85	1.76	17.53
6	1	–1	1	31.15	1.75	17.80
7	1	1	–1	31.56	1.77	17.83
8	1	1	1	27.39	1.50	18.26
9	-α	0	0	23.67	1.37	17.28
10	α	0	0	30.56	1.77	17.27
11	0	-α	0	25.15	1.41	17.84
12	0	α	0	24.11	1.36	17.73
13	0	0	-α	26.16	1.50	17.44
14	0	0	α	25.23	1.37	18.42
15	0	0	0	25.67	1.47	17.46
16	0	0	0	25.05	1.42	17.64

Table 3 shows the regression data for the P_2_O_5_/Fe_2_O_3_ response in phosphoric acid. The analysis of variance (ANOVA) employed a statistical significance of *p*-value ≤ 0.06. The R-squared and the adjusted R-squared were provided for the model, and the values signal that the model is suitable to represent the data and make predictions.

1 $$Y1=17.568+0.101X1-0.185{X1}^{2}+0.123{X2}^{2}+0.328X3+0.211{X3}^{2}+0.189X1X2-0.131X1X3$$

**Table 3:** Analysis of Variation (ANOVA) for P_2_O_5_/Fe_2_O_3_ Ratio Response for CCD 1 Experiments

term	sum of squares	*df*	mean square	*F*-value	*p*-value	B coefficient estimate	standard error coefficient
constant	-	-	-	-	0.00000	17.568	0.0076
X1	0.114	1	0.114	6.690	0.03229	0.101	0.0388
(X1)^2^	0.187	1	0.187	10.968	0.01067	–0.185	0.0558
(X2)^2^	0.083	1	0.083	4.868	0.05841	0.123	0.0558
X3	1.217	1	1.217	71.269	0.00003	0.328	0.0388
(X3)^2^	0.243	1	0.243	14.253	0.00542	0.211	0.0558
X1·X2	0.285	1	0.285	16.683	0.00351	0.189	0.0462
X1·X3	0.138	1	0.138	8.067	0.02180	–0.131	0.0462
residual	0.137	8	0.017	-	-	-	-
*R* ^2^ = 0. 943; *R* ^2^adj = 0.893							

Table 3 and eq 1 show that the response P_2_O_5_/Fe_2_O_3_ (Y1) increases linearly with the variables reaction temperature (X1), precipitation time (X2), and percentage mass of added reagent (X3). Among the analyzed variables, the greatest influence on the response of P_2_O_5_/Fe_2_O_3_ is related to the variable percentage mass of added reagent (X3), followed by its quadratic term of percentage mass of added reagent (X3^2^).

Based on the fit in eq 1, one can compare the observed and predicted values (Figure 2). The graph shows good accuracy and strong correlation between the model and the observed values, as the values are scattered within the ± 95% confidence level. The independent variables selected in this study had a significant effect on P_2_O_5_/Fe_2_O_3_ (Y1).

**Figure 2:**
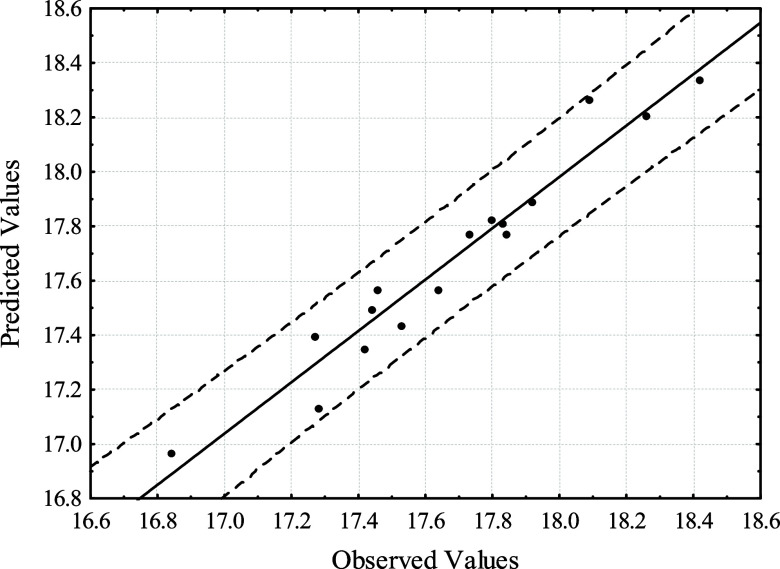
Comparison between observed and predicted values for the P_2_O_5_/Fe_2_O_3_ ratio in CCD 1 experiments. Dashed lines represent the 95% confidence interval.

The response surfaces of the P_2_O_5_/Fe_2_O_3_ ratio were generated using the prediction eq (eq 1), always analyzing two variables at a time and the missing variable at their central point (Figure 3). It is evident that different variables play a role in the reduction of iron oxides in phosphoric acid. In Figure 3a, where reaction temperature and precipitation time are varied while using the percentage of reagent mass as the central point variable, the surface profile exhibits a saddle-type behavior. The highest response values are observed at elevated reaction temperatures and prolonged precipitation times. Moving to Figure 3b, the surface demonstrates higher model response values for higher reagent percentages, emphasizing the greater influence of variable X3 over X1, consistent with eq 1. This trend persists in Figure 3c, where high values of variable X3 also result in the highest response values. This result is expected; as more reactions were added, the more iron oxides were removed from the system, and due to the low efficiency of reagent 1 associated with high iron contents from the acid, the samples did not reach a point where the reactant was in excess.

**Figure 3:**
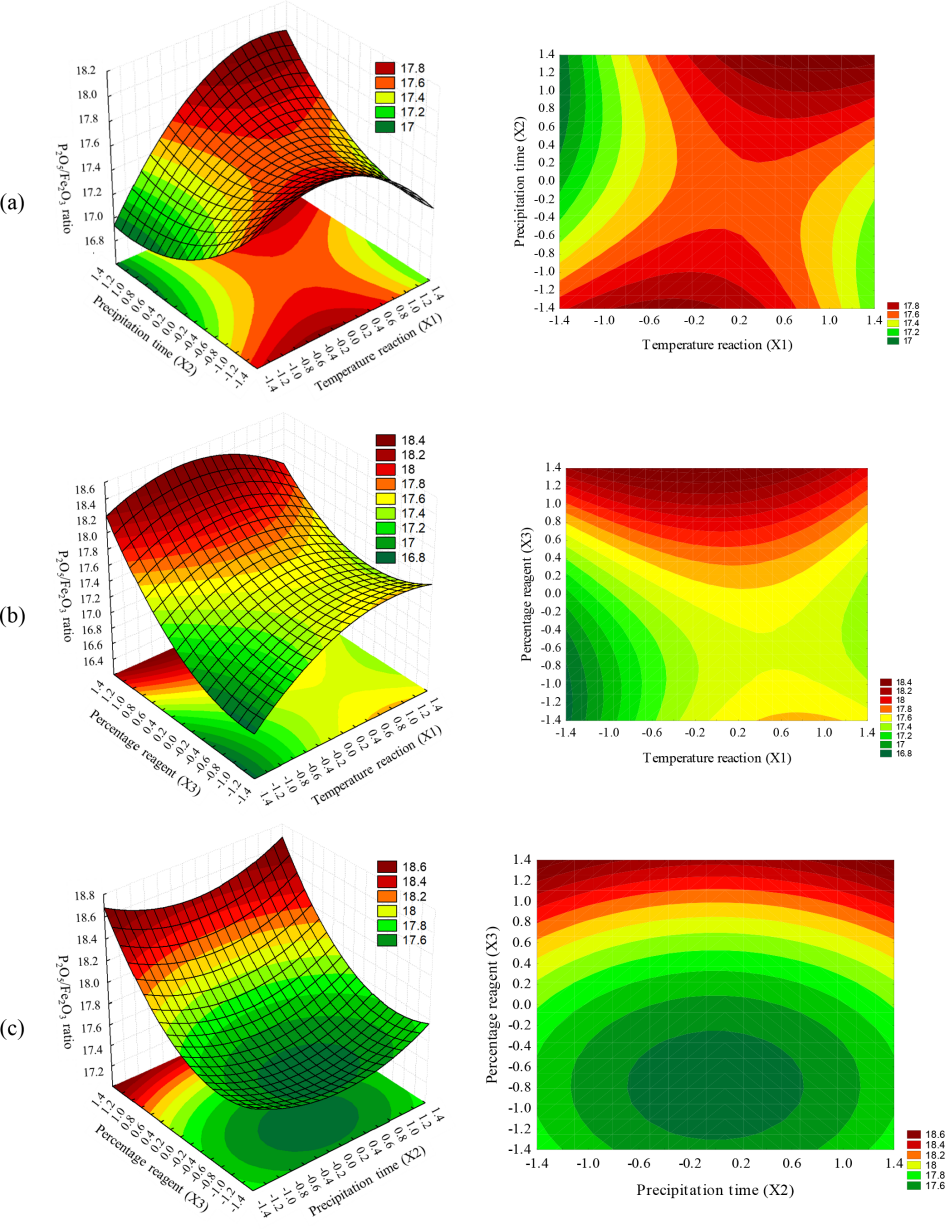
Plots for the P_2_O_5_/Fe_2_O_3_ ratio (3D on the left and 2D projection on the right)—CCD 1: (a) reaction temperature (X1) and precipitation time (X2) with percentage mass of added reagent (X3) at the central level; (b) reaction temperature (X1) and percentage mass of added reagent (X3) with precipitation time (X2) at the central level; and (c) precipitation time (X2) and percentage mass of added reagent (X3) with reaction temperature (X1) at the central level.

Overall, all of the variables under study had an influence on the reduction of the levels of iron oxides in phosphoric acid, although with little impact on the overall value of the variable. This characteristic is the result of the mixture of phosphonic acids and dispersants used, which combine their effects for a chelating effect. However, as each compound is in small concentrations in the product used, its effects are reduced when the objective is to reduce only one contaminant present in the acid. Such behavior is due to the presence of polyacrylates in the formulation since such compounds usually do not support high temperatures. Increments in variable X2, the precipitation time, generated small increases in the removal of iron oxides in the medium, being the variable that least influenced the system and showing that the precipitation reaction time has little influence on the main response. Also, it is expected that the polyacrylates will affect the chelated fraction of iron phosphates formed by phosphonic acids. If not enough chelated particles are formed, the reactant will not be effective on the soluble iron phosphates present on the phosphoric acid.

The phosphate and iron oxide contents on the solids precipitated generated in CCD 1 are shown in Table 4. The reagent used in fact increases the levels and masses of precipitated iron, aluminum, and magnesium oxides. There is a direct correlation between the increase in the mass of added reagent, variable X3, and the mass of precipitated solids formed on the tests. The direct comparison of runs 1 and 2, or 3 and 4, shows this mass increase on the same temperature (X1) and precipitation time (X2) levels. Analyzing the central points, the variable X2, precipitation time, reduced the masses generated from precipitates, indicating that part of the precipitates generated were resolubilized. The total precipitated masses are too low for the reagent to have an effective action in reducing the iron oxide contaminants present in the acid, especially at this stage of the process. To be effective, higher masses of precipitate should have been formed in this step, or a chelated material with higher iron oxide concentrations should have been verified.

**Table 4:** P_2_O_5_ and Fe_2_O_3_ Contents in the Precipitates Formed during CCD 1 Experiments

	variables			contents					masses (g)	
	X1	X2	X3	P_2_O_5_ (%)	Fe_2_O_3_ (%)	CaO (%)	Al_2_O_3_ (%)	MgO (%)	generated solids	Fe_2_O_3_
1	–1	–1	–1	3.29	2.04	28.32	0.02	0.14	4.21	0.09
2	–1	–1	1	10.93	4.48	11.63	0.11	0.17	13.24	0.59
3	–1	1	–1	2.6	1.9	30.01	0.03	0.01	4.8	0.09
4	–1	1	1	8.2	4.59	13.57	0.03	0.30	12.5	0.57
5	1	–1	–1	1.32	0.73	29.44	0.02	0.14	4.65	0.03
6	1	–1	1	3.98	2.31	15.42	0.07	0.14	10.81	0.25
7	1	1	–1	1.36	4.09	29.42	0.03	0.02	6.22	0.25
8	1	1	1	5.11	3.66	15.81	0.03	0.30	9.91	0.36
9	-α	0	0	4.19	2.01	19.68	0.03	0.04	11.66	0.23
10	α	0	0	2.22	0	24.52	0.03	0.09	11.01	0.00
11	0	-α	0	6.91	4.48	20.22	0.03	0.15	6.14	0.28
12	0	α	0	4.69	2.9	23.35	0.05	0.15	0	0.00
13	0	0	-α	1.21	0.85	31.66	0.03	0	5.11	0.04
14	0	0	α	7.75	4.15	14.01	0.03	0.35	13.23	0.55
15	0	0	0	4.99	3.69	21.21	0.03	0.15	7.65	0.28
16	0	0	0	5.67	4.11	21.38	0.03	0.11	7.03	0.29

However, this reagent can be effective as a scale inhibitor in concentration systems, since the precipitation of several metal oxides occurred, with a noticeable increase in MgO content on experiments 4, 8, and 14. Gypsum (CaSO_4_) is the main reaction product of the wet-process phosphoric acid production route and the expected product of the precipitation of diluted and filtered phosphoric acid. Therefore, the CaO content analysis shows that the main precipitate formed is gypsum (CaSO_4_), which becomes diluted as X3 increases the precipitate mass.

Table 5 presents the cake washing water analysis, with P_2_O_5_ and Fe_2_O_3_ contents. The analysis of the wash water contents demonstrates that the residual levels of iron oxides in the system are inexpressive and close to the quantification limits of the analytical method. This fact, therefore, demonstrates that basically all of the iron oxides remained with the acid or in the precipitates generated. The P_2_O_5_ analyses show that there were no filtration difficulties in any of the experiments, mainly due to the low precipitate mass generated and since most of the solids generated were dehydrated gypsum, with an adequate filtration rate on the leaf tests.

**Table 5:** P_2_O_5_ and Fe_2_O_3_ Contents of Washing Water from CCD 1 Experiments

	variables			contents	
	X1	X2	X3	P_2_O_5_ (%)	Fe_2_O_3_ (%)
1	–1	–1	–1	1.00	0.06
2	–1	–1	1	2.84	0.11
3	–1	1	–1	1.11	0.09
4	–1	1	1	3.41	0.07
5	1	–1	–1	1.45	0.1
6	1	–1	1	0.39	0.09
7	1	1	–1	1.27	0.08
8	1	1	1	2.65	0.08
9	-α	0	0	2.01	0.06
10	α	0	0	2.06	0.07
11	0	-α	0	2.12	0.05
12	0	α	0	1.87	0.07
13	0	0	-α	0.99	0.09
14	0	0	α	3.82	0.08
15	0	0	0	2.00	0.06
16	0	0	0	2.07	0.08

### 3.2 CCD 2—DTPMP 60% Solution

Table 6 presents the P_2_O_5_ and Fe_2_O_3_ contents on the phosphoric acid generated for all points of CCD 2, which used a 60% solution of diethylenetriamine penta (phosphonic methylene) as reactant. Analyzing the P_2_O_5_/Fe_2_O_3_ ratio, all tests resulted in an increase in relation to the reference test, indicating that the use of 60% DTPMP led to significant decreases in the iron content. Overall, by analyzing separately runs 1 and 5, 4 and 8, and 9 and 10, it can be observed that the variation in the values of the reaction temperature variable (X1) did not alter the P_2_O_5_/Fe_2_O_3_ response in phosphoric acid, a positive outcome when considering the need to control the temperature on the industrial reactors. Looking at runs 6 and 8, a slight variation in the P_2_O_5_/FeO_3_ ratio is noted when the precipitation time values (X2) are altered. It is evident that the variable that most influenced the removal of iron oxides was the mass of reactant added, represented by coded variable X3. This variation can be observed when analyzing runs 1 and 2, as well as runs 3 and 4. The use of higher quantities of the reagent 2 led to very good results, as can be observed in runs 2, 4, and 14. The best condition using the additive 60% DTPMP was obtained in run 14, with a removal of 40% of Fe_2_O_3_ on a dry basis, leading to a P_2_O_5_/Fe_2_O_3_ ratio of 26.04 in the phosphoric acid. The total Fe_2_O_3_ removal achieved was 0.58% on a dry basis, the highest removal currently achieved on a diluted phosphoric acid from igneous phosphate concentrates, and a value comparable to other works utilizing other reagents as chelating agents for sedimentary phosphates phosphoric acid.^26^

**Table 6:** P_2_O_5_ and Fe_2_O_3_ Contents on Phosphoric Acid with the Addition of 60% DTPMP (Reagent 2)

CCD 2	variables			response		
	X1	X2	X3	P_2_O_5_ (%)	Fe_2_O_3_ (%)	P_2_O_5_/Fe_2_O_3_
reference	-	-	-	23.86	1.46	16.34
1	–1	–1	–1	24.83	1.40	17.74
2	–1	–1	1	23.95	1.02	23.48
3	–1	1	–1	24.35	1.35	18.04
4	–1	1	1	23.51	0.94	25.01
5	1	–1	–1	26.85	1.44	18.65
6	1	–1	1	27.49	1.20	22.91
7	1	1	–1	30.05	1.68	17.89
8	1	1	1	25.76	1.06	24.30
9	-α	0	0	23.36	1.15	20.31
10	α	0	0	31.57	1.48	21.33
11	0	-α	0	24.86	1.24	20.05
12	0	α	0	24.85	1.25	19.88
13	0	0	-α	25.91	1.47	17.63
14	0	0	α	24.22	0.93	26.04
15	0	0	0	25.51	1.24	20.57
16	0	0	0	25.59	1.23	20.80

Several experimental points presented a P_2_O_5_/Fe_2_O_3_ ratio of >20, a common benchmark required for adequate MAP production. The central points also reached this minimum requirement. As per the experimental design, the central points were based on the standard clarification temperature and settling time already used industrially, which means that no process changes besides adding the chelating agent are necessary to reach product specifications when using 60% DTPMP with the reference phosphoric acid. Data also showed that higher ratios are achievable, which means that the route can potentially enable the usage of phosphate concentrates with higher iron contents, debottlenecking mining operations or mineral processing operations.

The effects of variables, reaction temperature (X1), precipitation time (X2), and percentage mass of added reagent (X3), on P_2_O_5_/Fe_2_O_3_ (Y2) were analyzed by regression (Table 7) and represented by eq 2. By the analysis of each coefficient, it is possible to verify which variables have more effect on the response and if these contributions were due to the linear term, the quadratic term or the interaction terms. The signal of each coefficient demonstrates whether the term increases or decreases the response. It is verified through the coefficients of determination (*R* ^2^), 0.978, that the results obtained were adequately represented by the mathematical models created. The parameters of eq 2 were statistically significant (*p*-value ≤ 0.06). Statistical analysis indicated that the residues are normal and independently distributed with a mean of zero and a constant variance.

2 $$Y2=20.745-0.445{X2}^{2}+3.023X3+0.684{X3}^{2}+0.423X2X3$$

**Table 7:** Analysis of Variation (ANOVA) for P_2_O_5_/Fe_2_O_3_ Ratio Response in CCD 2 Experiments

term	sum of squares	*df*	mean square	*F*-value	*p*-value	B coefficient estimate	standard error coefficient
constant	-	-	-	-	0.00000	20.745	0.234
(X2)^2^	1.085	1	1.085	4.845	0.04998	–0.445	0.202
X3	103.414	1	103.414	461.734	0.00000	3.023	0.141
(X3)^2^	2.569	1	2.569	11.471	0.00607	0.684	0.202
X2.X3	1.428	1	1.428	6.376	0.02822	0.423	0.167
residual	2.464	11	0.224	-	-	-	-
*R* ^2^ = 0. 978; *R* ^2^adj = 0.969							

The data presented in Table 7 shows that term X1 and the interaction between this variable with the others were not statistically significant when considering the *p*-value <0.06. The R-squared and the adjusted R-squared were provided for the model, and the values signal that the models are suitable to represent the data and to make predictions. The independent variables coded, X2 and X3, had a significant effect on P_2_O_5_/Fe_2_O_3_ (Y2). The only significant interaction effects were those that included variable X3 (percentage mass of added reagent). Abdel-Ghafar analyzed the process of purification of high-iron wet-process phosphoric acid via the oxalate precipitation method.^19^ The authors also verified that the clarification time and oxalic acid dose had a significant effect on removing iron.

Starting from the regression adjustment for the P_2_O_5_/Fe_2_O_3_ response, the comparison between the observed and predicted values is shown in Figure 4. The graph demonstrates good accuracy and a strong correlation between the model and the observed values, as the values are scattered within the ±95% confidence level.

**Figure 4:**
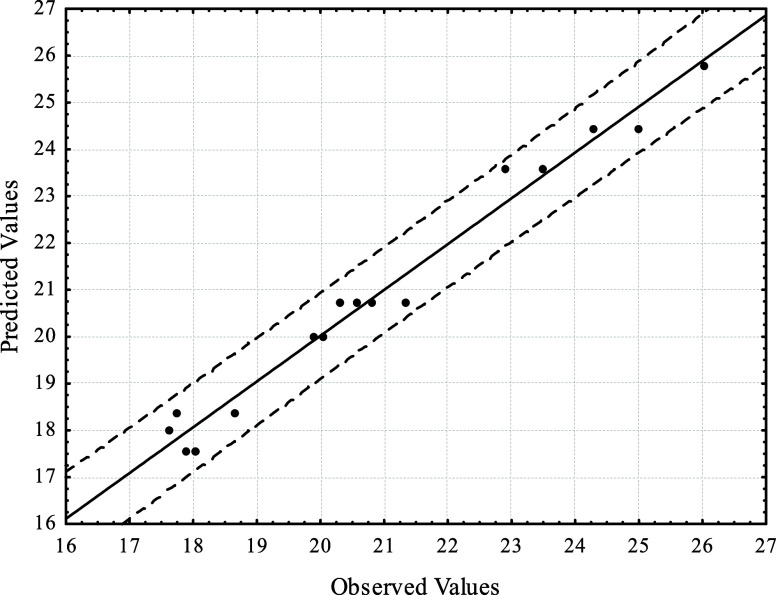
Comparison between observed and predicted values for the P_2_O_5_/Fe_2_O_3_ ratio in CCD 2 experiments. Dashed lines represent the 95% confidence interval.

Using the obtained quadratic models, response surface 3D and contour plots were plotted to map the system behavior as a function of the variables levels (Figure 5). This response surface was obtained from the prediction eq (eq 2). Figure 5a shows the response surface of the P_2_O_5_/Fe_2_O_3_ ratio as a function of reaction temperature (X1) and precipitation time (X2) with the percentage mass of added reagent (X3) at the central level. One can observe a slight variation in the response across the entire range of X1 and X2 evaluated, ranging from approximately 20 to 20.6. As expected by the fitting equation, the P_2_O_5_/Fe_2_O_3_ ratio is not altered by the change in reaction temperature. For Figure 5b, as observed previously, there was no variation in the P_2_O_5_/Fe_2_O_3_ ratio in the evaluated temperature range. However, it is observed that iron removal is favored by increasing the amount of reagent added to the reaction system. The observed effect is beneficial industrially, as there would be no need for changes in the process of stream temperatures in the event of potential industrial use.

**Figure 5:**
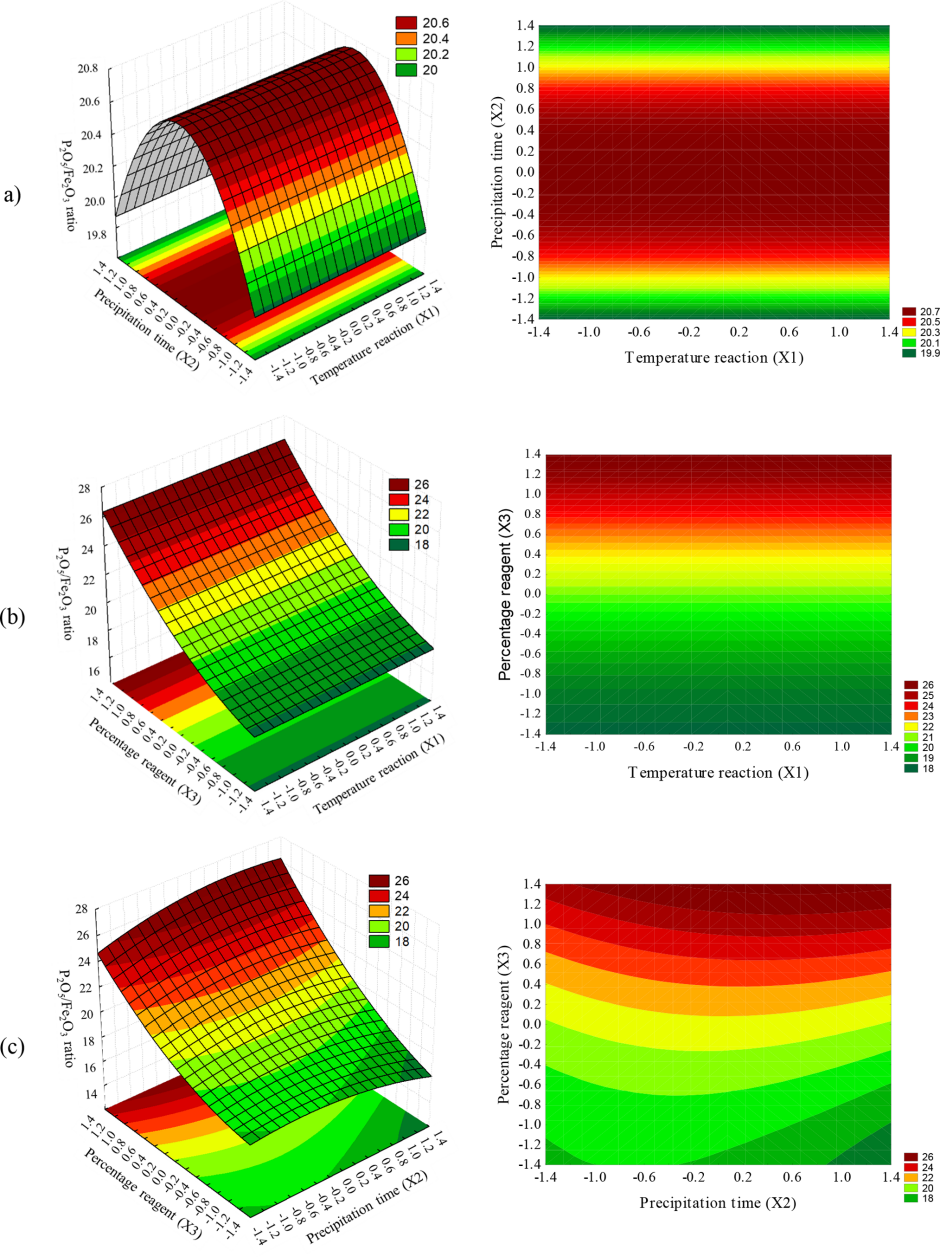
Plots for P_2_O_5_/Fe_2_O_3_ ratio (3D on the left and 2D projection on the right)—CCD 2 using 60% DTPMP (reagent 2): (a) reaction temperature (X1) and precipitation time (X2) with percentage mass of added reagent (X3) at the central level; (b) reaction temperature (X1) and percentage mass of added reagent (X3) with precipitation time (X2) at the central level; and (c) precipitation time (X2) and percentage mass of added reagent (X3) with reaction temperature (X1) at the central level.

The variable that most influenced the removal of iron oxides was the mass of the chelating agent added, represented in the coded variable X3 and expressed in the response surfaces. Such behavior is expected since greater additions of reagent benefit the iron oxide precipitation capacity. In precipitation times close to the region of level +α in X2, there is a considerable increase on the P_2_O_5_/Fe_2_O_3_ ratio with the increase in the added reagent mass, indicating that there was a greater precipitation of iron oxides.

The variable X2, precipitation time, exerted a positive influence on iron removal. In addition, the interaction between variables X2*X3 showed an increase characteristic in the P_2_O_5_/Fe_2_O_3_ ratio, indicating that iron removal was benefited with simultaneous increments of both variables. Possibly, longer precipitation times would increase the response, since, when analyzing the samples of phosphoric acid with precipitation powders, they are indicative that the reactions of 60% DTPMP (reagent 2) with iron oxides are not instantaneous and that they continue even several hours after adding the reagent. However, industrial units do not have residence times in their clarification circuits to make times longer than the studied range feasible. Song et al. analyzed the precipitation of iron as jarosite by using a combination process of electrolytic reduction and biological oxidation.^27^ The authors verified that the system reached a steady state at 96 h; that is, a longer time than would be feasible without industrial changes to increase residency time. For this time, the removal efficiency of soluble iron in the solution no longer increased, even if there is an increase of the reaction time.

After the reaction process, the iron content in the phosphoric acid was analyzed, as presented earlier, and the quantities of precipitated iron and in the wash and water were also quantified. Table 7 presents the data of P_2_O_5_ and Fe_2_O_5_ retained in the precipitates as a percentage and in terms of the mass of precipitate generated in each experiment. The precipitates generated were analyzed as described in the methodology. With only the contents of the generated precipitates, it is not possible to quantify the efficiency of removing the oxides since each experiment generated a different mass of precipitate. Thus, Table 7 also presents the mass of precipitate generated in each experiment, as well as the mass of each precipitated oxide, calculated according to the levels of each analysis. It should be noted that all masses were measured on a dry basis so that the residual moisture of each cake did not influence the masses.

Runs 1 and 2, 3 and 4, and 5 and 6 (Table 8) indicate that an increase in the amount of the 60% DTPMP reagent results in a higher content and greater mass of precipitated iron oxides. Corroborating the results in Table 6, the higher the iron oxide mass on the precipitate, the lower the iron oxide content of the phosphoric acid.

**Table 8:** P_2_O_5_ and Fe_2_O_3_ Contents in the Precipitates Formed with the Addition of 60% DTPMP (Reagent 2) to Phosphoric Acid

	variables			contents					masses (g)	
	X1	X2	X3	P_2_O_5_ (%)	Fe_2_O_3_ (%)	CaO (%)	Al_2_O_3_ (%)	MgO (%)	generated solids	Fe_2_O_3_
1	–1	–1	–1	5.29	3.42	16.15	0.25	0.27	10.00	0.34
2	–1	–1	1	8.54	4.30	18.29	0.45	0.38	28.01	1.20
3	–1	1	–1	15.35	6.60	15.80	0.32	0.24	10.64	0.70
4	–1	1	1	13.08	9.09	9.31	0.43	0.37	24.98	2.27
5	1	–1	–1	13.74	2.11	6.69	0.43	0.36	8.64	0.18
6	1	–1	1	11.85	3.30	6.32	0.51	0.36	29.83	0.98
7	1	1	–1	11.53	5.72	17.11	0.39	0.32	8.76	0.50
8	1	1	1	19.50	9.74	5.87	0.48	0.22	22.43	2.18
9	-α	0	0	10.54	3.71	10.64	0.47	0.37	15.75	0.58
10	α	0	0	11.03	3.11	11.72	0.54	0.38	16.01	0.50
11	0	-α	0	13.43	6.29	4.57	0.55	0.38	15.15	0.95
12	0	α	0	12.23	6.83	12.91	0.52	0.38	17.27	1.18
13	0	0	-α	11.58	5.44	7.74	0.50	0.37	35.48	1.93
14	0	0	α	22.21	9.14	4.25	0.40	0.18	31.62	2.89
15	0	0	0	11.84	4.56	7.89	0.57	0.39	14.6	0.67
16	0	0	0	12.30	4.98	9.30	0.59	0.33	14.74	0.73

The precipitates contain levels of calcium oxides in the form of precipitated gypsum as well as aluminum and magnesium oxides, and even though they represent a very small mass of the overall precipitate, a correlation can be seen when comparing the total mass precipitated with higher contents of MgO and Al_2_O_3_. By the reaction mechanism of organic phosphonic acids, it is known that precipitation of P_2_O_5_ will occur in the form of iron phosphonates and other phosphonates, in such a way that such precipitation will be proportional to the mass of Fe_2_O_3_ precipitated.^28^

The analysis of the washing content, as presented in Table 9, shows that the residual iron oxide levels in the system are inexpressive and close to the quantification limits of the analytical method. This fact, therefore, demonstrates that basically all iron oxides remained with the acid or in the precipitates generated. There was a clear increase in the P_2_O_5_ content of the water in the experiments that generated a greater mass of precipitate since greater masses of cake in the filter absorb greater proportional masses of phosphoric acid. However, even in experiments where there was an increase in residual P_2_O_5_ levels in the washing water, such levels did not exceed the typical content of recycled phosphoric acid streams in industrial units. Such content is expected and is industrially recovered on the gypsum washing phases.

**Table 9:** P_2_O_5_ and Fe_2_O_3_ Contents Washing Water from CCD 1 Experiments

	variables			contents	
	X1	X2	X3	P_2_O_5_ (%)	Fe_2_O_3_ (%)
1	–1	–1	–1	1.50	0.07
2	–1	–1	1	6.22	0.07
3	–1	1	–1	1.11	0.09
4	–1	1	1	0.88	0.05
5	1	–1	–1	3.19	0.07
6	1	–1	1	7.17	0.07
7	1	1	–1	0.32	0.06
8	1	1	1	6.08	0.08
9	-α	0	0	4.41	0.06
10	α	0	0	5.92	0.07
11	0	-α	0	3.70	0.06
12	0	α	0	4.09	0.06
13	0	0	-α	6.81	0.08
14	0	0	α	5.98	0.11
15	0	0	0	0.46	0.08
16	0	0	0	3.60	0.07

The precipitated solids, when filtered under experimental conditions mainly on runs that formed more precipitate or removed greater masses of iron oxides, presented low filtration rates, which are unfeasible for an industrial process. As a rule, the higher the P_2_O_5_ content of the wash water, the lower the filtration rate was. Such filtration rates could be increased with the use of flocculant polymers or with the mixture of the generated solids stream with the gypsum formed in the reactor. The industrial conceptual design of these experiments consists of mixing the cake formed on the reactions with the gypsum, so the cake can be washed and the particle size from the dihydrate calcium sulfate can increase the filtration rate.

The precipitate mass generated in the experiments is low compared to the gypsum mass generated on attack units. While 1 ton of P_2_O_5_ in the attack system generates approximately 4.6 tons of gypsum,^29^ the treatment proposed on CCD2 would generate 200 kg of extra solids in its most aggressive scenario. Such solid mass, when mixed with gypsum, does not affect the filtration rate of the system in any considerable way. However, the added mass could reduce the capacity of industrial filters due to increments in gypsum cake height, which would rarely consist of a limitation. To prove this scenario, the following images showcase the analysis done to evaluate if the production unit would lose filtration rate when implementing the proposed route. Figure 6a presents the solids generated after the reaction and filtration, with compositions as described in Table 8, while Figure 6b shows a sedimentation test of the solids formed during the reaction with phosphoric acid after 30 h of settling, showing that the chelated solids are not easily removable to generate a clarified acid. To increase the filtration rate on the chelated solids medium, several flocculant polymers were tested, and Figure 7 provides a comparison of different flocculant polymers used to flocculate the solids, effectively solving the acid clarification issue when upscaling the process. Finally, Figure 8 displays a microscopic picture of the mixture of the chelated iron phosphates with gypsum crystals in a scenario where formed solids are discharged on the gypsum filter, where the large needle-like crystals represent calcium sulfate dehydrate, and the smaller particles correspond to the chelated solids in the cake. This condition allowed filtration rates equivalent to the observed flow rates on the typical industrial production. Therefore, the chelated material can be stacked and incorporated into the gypsum, leading to a minimal impact on the gypsum handling operation.

**Figure 6:**
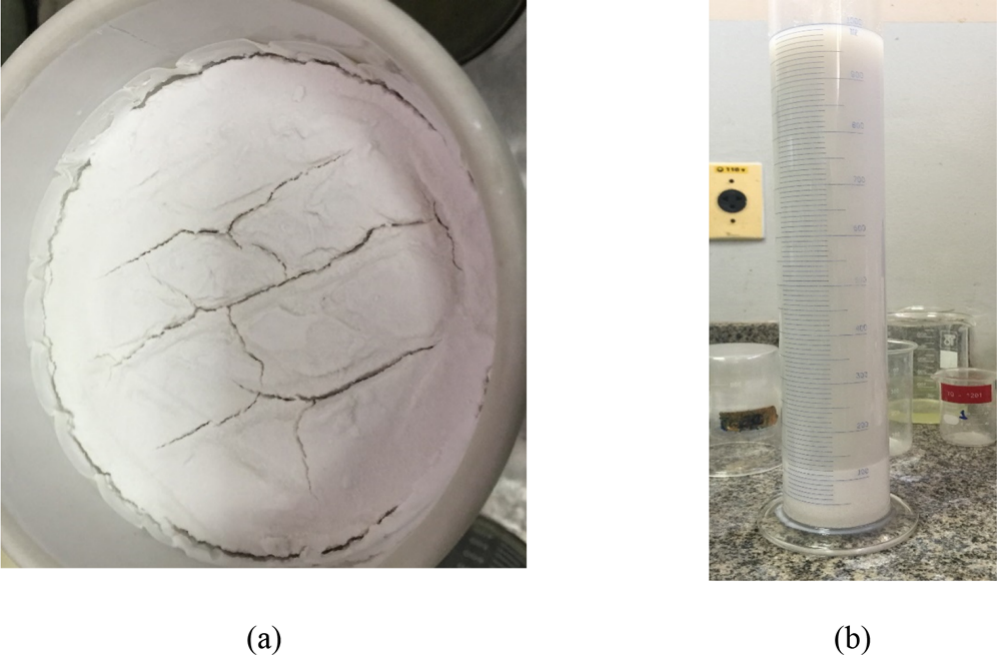
(a) Solids generated (cake) after filtration and (b) sedimentation test of the solids formed after 30 h. Photographs courtesy of Gustavo P. Ribeiro, Copyright 2025.

**Figure 7:**
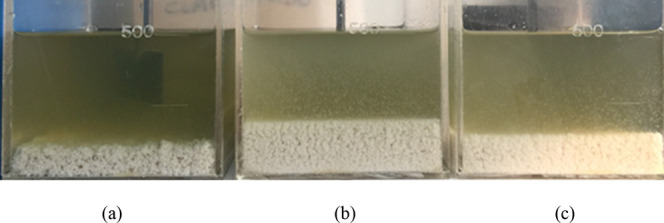
Comparison of different flocculants after 30 s: (a) high molecular weight anionic polymer, (b) medium molecular weight anionic polymer, and (c) low molecular weight anionic polymer. Photographs courtesy of Gustavo P. Ribeiro, Copyright 2025.

**Figure 8:**
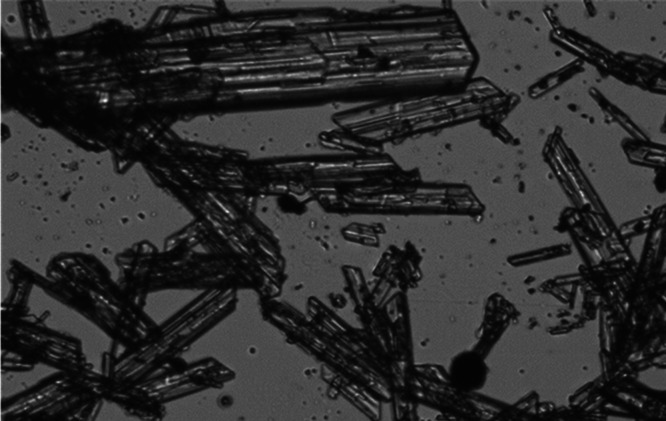
Generated solids mixed in proportion with gypsum for leaf test.

Comparing the central points, runs 15 and 16 from Tables 2 and 6, it is noted that, overall, the use of reagent 1, polyacrylates associated with phosphonic acids, was less efficient in removing iron from phosphoric acid compared to reagent 2, 60% DTPMP, as the variations observed in the P_2_O_5_/Fe_2_O_3_ ratio were higher than those seen in Table 2. Unlike what was observed for reagent 2, when reagent 1 was used, a variation in iron removal was noted when the reaction temperature was altered; for example, comparing runs 1 and 2, 3 and 4, and 5 and 6. When analyzing runs 1 and 3, 2 and 4, and 5 and 7, it is observed that the effect on iron removal from variable X2, precipitation time, is very small.

Based on the results obtained, it is evident that the choice of 60% DTPMP reagent significantly impacts the removal of iron oxides from phosphoric acid solutions. Such a reduction could increase the mine’s lifespan, as phosphate rocks with higher iron contents could be industrially used. A reduction of 0.58% in iron oxides in the acid could allow the usage of phosphate concentrates with an approximately 2% higher content of iron oxides.

The distinct performance observed between the two reagents can be attributed to their different modes of interaction with iron species in phosphoric acid. In the case of the DTPMP solution, the removal of Fe_2_O_3_ likely occurs via chelation, with the phosphonic functional groups forming stable, soluble complexes with Fe^3+^ ions. This mechanism is supported by analogous reactions described for aminomethylene phosphonic acids, such as ATMP, in which iron–phosphonate complexes are formed and phosphoric acid is released as a byproduct, potentially contributing to an increase in P_2_O_5_ availability. Molecular analyses and adsorption studies confirm the strong affinity of organic phosphonic groups in DTPMP for metal ions, which enhances the iron removal efficiency.^30^ In contrast, the mixture of polyacrylates and phosphonic acids appears to act primarily through particle dispersion, possibly stabilizing iron-containing colloids and limiting agglomeration with a less pronounced complexation effect under the highly acidic conditions of the medium. This dual mechanism, combining dispersive action and low-affinity complexation, may explain the lower iron removal efficiency and higher variability observed with this reagent.

Both reactants form chelates when in contact with phosphoric acid under conditions where the active ingredient fully reacts on the analyzed reaction times. As they are water-based solutions, the water and other unreacted compounds are carried over to the diluted phosphoric acid. These compounds have no significant impact on phosphoric acid or the fertilizers produced. Due to the low mass of reactant added and to the chelation of iron oxides and other compounds, the P_2_O_5_ content on the acid increases on most runs, a result that reduces the energy required to concentrate the phosphoric acid when comparing those experiments with the reference, which was produced using the central points of all variables.

From a financial standpoint, the usage of chelating agents adds from 0.03 to 0.1 USD per kilo of chelated phosphoric acid. It can be a significant cost to operations that produce millions of tons of sulfate per year. Its usage, however, should be defined on a basis-by-basis considering the Fe_2_O_3_ content present in the phosphoric acid and the ability to consistently reach the final product specifications on the fertilizers.

## 4 Conclusion

This study investigated two different reagents for the removal of iron from phosphoric acid, aiming to improve its quality for fertilizer production. The use of diethylenetriamine penta (methylene phosphonic) (DTPMP) and polyacrylates associated with phosphonic acids showed promising results, with DTPMP being more effective in reducing iron content. The experimental designs, regression analyses, and response surface methodologies provided valuable insights into the effects of the reaction temperature, precipitation time, and reagent dosage on iron removal efficiency. Furthermore, the analysis of the wash water content highlighted the effectiveness of the removal process, with residual iron oxide levels being negligible. Upon analyzing the experimental designs, an increase in the response values, P_2_O_5_/Fe_2_O_5_ ratio, is noted in both cases, the mixture of polyacrylates associated with phosphonic acids and the solution with 60% diethylenetriamine penta (phosphonic methylene) (60% DTPMP), compared to the reference runs. For CCD 1, there were increases in responses ranging from 3 to 12% compared with the reference value. As for CCD 2, these values varied from 7 to 59% compared to those of the reference. In both CCDs, the optimal condition was observed in the run using *T* = 55 °C, a precipitation time of 14 h, and 5.57% of the mass percentage of reagent. The best results obtained in CCD 2 indicate that the choice of the 60% DTPMP reagent significantly impacts the removal of iron oxides from phosphoric acid solutions. The industrial use of a solution with 60% of diethylenetriamine penta (phosphonic methylene) (60% DTPMP) can lead to higher quality phosphoric acid and improve the use of phosphate rocks with high iron oxides contents, impacting mine’s lifespan and operations.
